# Unpacking the COVID-19 rescue and recovery spending: an assessment of implications on greenhouse gas emissions towards 2030 for key emitters

**DOI:** 10.1007/s44168-022-00002-9

**Published:** 2022-03-07

**Authors:** Frederic Hans, Santiago Woollands, Leonardo Nascimento, Niklas Höhne, Takeshi Kuramochi

**Affiliations:** 1 NewClimate Institute, Cologne, Germany; 2 Willy Brandt School of Public Policy, University of Erfurt, Erfurt, Germany; 3 Environmental Systems Analysis Group, Wageningen University, Wageningen, The Netherlands; 4 Copernicus Institute of Sustainable Development, Utrecht University, Utrecht, The Netherlands

**Keywords:** Green recovery, Fiscal stimulus, COVID-19, Climate change mitigation, Environmental economics

## Abstract

This paper analyses how fiscal stimulus spending in response to the COVID-19 pandemic supports the low-carbon transition. We developed a new framework to categorise rescue and recovery spending measures according to their level of greenness and their type of expected impact on greenhouse gas emissions. This framework allows to better capture how measures’ emission impacts may unfold over time and to identify the share of fiscal spending missing robust conditions or incentives to be considered low carbon. We assess nearly 2500 measures announced by 26 emitters as of May 2021, representing around 67% of global GHG emissions excluding land use in 2019. Our findings show that the largest share (35%) of spending with potential GHG emission implications went to measures that cannot be explicitly coded as high-carbon or low-carbon but substantiate current business-as-usual practice (‘supporting the status quo’). Our assessment reveals the different magnitudes to which the emitters have missed the opportunity for a green recovery. Low-carbon spending is sizeable (22%) across countries. However, almost two-thirds will likely rather unfold its impact over time. This fiscal spending may trigger emissions reductions through enabling or catalytic causal effects over time but will not necessarily lead to direct emission reduction impacts before 2030.

**Supplementary Information:**

The online version contains supplementary material available at 10.1007/s44168-022-00002-9.

## Introduction

Governments’ existing greenhouse gas (GHG) emissions reduction targets up to 2030 (Nationally Determined Contributions: NDCs) collectively remain unambiguously inadequate to put the world on an emissions pathway aligned with the Paris Agreement’s ‘well below 2 °C’ and 1.5 °C temperature limit (den Elzen et al., 2021; Höhne et al., 2020a; UNEP, 2020). The Paris Agreement requests countries to update their NDCs every 5 years, with the first update round taking place in 2020 (UNFCCC, 2015, Art. 4.9). The ratchet mechanism underlying the Paris Agreement expects each country to strengthen their GHG emissions reduction targets in the 2020 and in subsequent updates every 5 years as a “progression beyond the Party’s then current nationally determined contribution and reflect its highest possible ambition […] in the light of different national circumstances” (UNFCCC, 2015, Art. 4.3).

The fiscal rescue and recovery spending in response to the COVID-19 pandemic coincided with the first NDC update round. Lack of progress to achieve NDC targets and accelerate climate action in the last decade has increased the challenge to keep the temperature goals of the Paris Agreement within reach (Höhne et al., 2020a). Against this backdrop, the accelerated transition towards a global low-carbon economy mandates full alignment of all fiscal spending with the Paris Agreement goals. The Paris Agreement requires Parties to “make finance flows consistent with a pathway towards low greenhouse gas emissions and climate-resilient development” to achieve the Paris temperature goals (UNFCCC, 2015, Art. 2.1c and 2.1a).

Since the beginning of the COVID-19 pandemic, many governments worldwide have pledged to steer their rescue and recovery spending towards a ‘green’ recovery (O’Callaghan, 2021; Petersberg Climate Dialogue XI, 2020), often citing numerous economic, social, and environmental benefits of low-carbon spending (Höhne et al., 2020b). Since March 2020, an increasing body of literature have studied rescue and recovery spending by national governments and the potential effect of these measures on emissions pathways.

A first strand of literature conducts a variety of ex-ante assessments of different fiscal spending possibilities that do not evaluate on the emissions effects of these measures (Hepburn et al., 2020; IEA, 2020; Jotzo et al., 2020; Krebel et al., 2020). They aim to identify synergies between different economic, social, and environmental priorities by national governments. This strand of literature provides theoretical performance assessments of different policy types against pre-defined criteria. Where country-specific circumstances have been analysed, the assessments also provided recommendations on what to spend on and how much.

A second strand of research examines different emissions scenarios using stylised assumptions on the pandemic’s duration and intensity, and a hypothetically assumed carbon intensity of governments’ fiscal responses (Buckle et al., 2020; Climate Action Tracker, 2020; Forster et al., 2020; IEA, 2020; Lahcen et al., 2020; Meles et al., 2020; Pollitt et al., 2020; Shan et al., 2021). These studies do not analyse individual measures in detail but estimate the time it will take for the world (or selected economies) to recover or surpass pre-pandemic GDP levels and how different compositions of rescue and recovery packages can influence the recovery’s speed and carbon intensity. Most studies provide individual estimates of GDP and GHG emissions growth under stylized assumptions of ‘traditional stimulus’ and ‘green stimulus’ over different timeframes, ranging from 2024 (Shan et al., 2021) to 2050 (Forster et al., 2020).

These first two literature strands both represent important analyses to conceptually understand potential impacts of fiscal rescue and recovery spending ex-ante and to guide policy makers in the design and decisions phase. However, they do not evaluate the actual fiscal spending undertaken by governments.

A third strand tracks and assesses incoming rescue and recovery packages by national governments worldwide ex-post. These trackers categorise rescue and recovery measures in terms of their expected net GHG effect across different countries (E3G & Wuppertal Institut, 2021; Energy Policy Tracker, 2021; IEA, 2021c; O’Callaghan et al., 2021b; OECD, 2021; Vivid Economics, 2021). They often have different scopes in terms of country coverage, measure coverage, and method to differentiate between low-carbon and high-carbon measures. These frameworks generally classify policy archetypes into different groups based on their expected contribution to mitigation outcomes, and in some cases include other socio-economic considerations such as short and long-run multipliers, impacts on air pollution, and others.

This literature provides insights into the size and spending patterns of rescue and recovery packages but have only provided limited assessment on the spending’s implications on GHG emissions over time. For example, the *Oxford Global Recovery Observatory* classifies fiscal measure archetypes according to their short-term and long-term emissions impacts (O’Callaghan et al., 2021b). The assessment of rescue and recovery packages of the 50 largest economies shows that only a few countries such as France or South Korea have a positive net impact in reducing GHG emissions (O’Callaghan et al., 2021).

These existing frameworks under the third literature strand face two limitations for systematically tracking incoming fiscal rescue and recovery measures. First, a categorisation of how these measures differ in terms of the causal relationship between the adoption of these measures (e.g. a low-carbon measure) and the associated emission reductions or increases over time (e.g. direct emission reduction impact) remains outside of their scope. This gap in existing analyses limits a more systematic overview on how fiscal pending breaks down by their causal effect on GHG emissions. Second, none of the trackers’ frameworks systematically identify measures that could have become low-carbon in nature if governments would have implemented robust low-carbon conditions or incentives alongside them. Such identification of measures that cannot be explicitly coded as high-carbon or low-carbon but substantiate current business-as-usual practice (‘supporting the status quo’) would allow for a more nuanced assessment of a missed opportunity for a green recovery by key emitters.

Against this backdrop, this study contributes to existing literature of the third strand by providing a more refined methodological framework and its subsequent application guided by the following research question: *How does fiscal rescue and recovery spending in response to the COVID-19 pandemic differ in terms of their causal effect on GHG emissions implications*? We categorise rescue and recovery spending measures according to both their level of ‘greenness’ and their type of expected impact on GHG emissions. We narrowly define the level of ‘greenness’ categorisation to capture the expected net GHG effect of a particular measure, not considering wider environmental impacts as for example done by Vivid Economics (2021). The framework allows to better capture how measures’ emission impacts may unfold over time and to identify the share of fiscal spending missing robust conditions or incentives to be considered low carbon. Outside the scope of this analysis, we do not assess the actual or likely impacts of the spending measures in terms of relative magnitude of contributions to transformational change or absolute GHG emission reductions.

We systematically analyse 16 Member States of the European Union (EU)^1^ and 10 other selected key emitters (Brazil, China, India, Indonesia, Japan, Saudi Arabia, South Africa, South Korea, the United Kingdom (UK), and the United States of America (USA)) as of May 2021. The country selection focused on G20 members considering geographical representation across different continents. These countries together accounted for around 67% of global GHG emissions excluding land use in 2019 (Gütschow et al., 2021) and represented around 77% of the world’s GDP in 2019 (World Bank, 2021). Data availability determined the selection of the 16 EU Member States (out of 27 EU Member States in total). This selection covers all G20 members within the EU (Germany, France, Italy) and other larger economies (Spain, Poland), representing 85% of the EU’s GHG emissions excluding land use in 2019 (Gütschow et al., 2021).

This article is structured as follows. The ‘Methods and data’ section describes the methods and data used for the analysis. We introduce a novel framework to categorise *emission impact type* of COVID-19 rescue and recovery measures to complement the level of greenness categorisation used in the literature. We then apply this framework to a harmonised dataset of almost 2500 rescue and recovery measures in the period between January 2020 and May 2021. The ‘Results and discussion’ section presents and discusses the findings of the analyses on the composition of fiscal rescue and recovery spending in response to the COVID-19 pandemic for according to this framework. Finally, conclusions and policy recommendations are drawn in the ‘Conclusions and recommendations’ section.

## Methods and data

### Categorisation of fiscal rescue and recovery measures by their level of greenness and their emission impact type

#### Level of greenness categorisation

We use the level of greenness categorisation previously applied in Höhne et al. (2020b). The framework categorises rescue and recovery measure as low-carbon, high-carbon, unclear, and neutral (see Table 1 for explanations for each measure category).

**Table 1:** Definitions of the level of greenness categories for fiscal rescue and recovery measures previously used in Höhne et al. (2020b) and further extended in this study

Category	Explanation	Stylised example
‘Low-carbon’	The measure triggers investment in low-carbon technologies or supports further advancement of such technologies through R&D or regulatory changes.	Direct investments in new renewable electricity generation capacity
‘Supporting the status quo’	The measure cannot be explicitly coded as high-carbon or low-carbon but substantiate current business-as-usual practice in the given country context. Such measures would have presented the opportunity for policy makers to implement accompanying distinct conditions for a low-carbon transition coupled to the respective fiscal rescue and recovery spending item. We use the subsequent coding rules: • The respective measure archetype cannot be explicitly coded high-carbon or low-carbon. • If the government would have introduced specific (mandatory) conditions on low-carbon transition or prioritisation of low-carbon products or services, this would have changed the measure’s coding to low-carbon.	Corporate airline bailouts without conditions for net zero transition or general VAT reductions without prioritisation of low-carbon products
‘High-carbon’	The measure triggers investments in new carbon-intensive technologies (e.g. investment in new coal plants) or supports the further advancement of such technologies through R&D or regulatory changes.	Direct investments in new coal-based electricity generation capacity
‘Neutral’	The measure has no or limited impact on emissions.	Health care or social-related spending or R&D for health-related innovations.
‘Unclear’	No expert judgement remains possible given the lack of available information.	Fiscal budget item for which a government does not disclose any further specific information at the time of analysis.

Our study further introduces an additional ‘supporting the status quo’ category. This category comprises measures that cannot be explicitly coded as high-carbon or low-carbon but substantiate current business-as-usual practice in the respective country context (e.g. corporate airline bailouts without conditions for net zero transition). Such measures would have presented an opportunity for policy makers to implement accompanying distinct conditions for a low-carbon transition coupled to the respective fiscal rescue and recovery spending item.

The level of greenness for each ‘supporting the status quo’ measure highly depends on the emissions intensity of current practice in the respective country and sector. This measure category therefore differs from most of the low-carbon and high-carbon measure archetypes (e.g. a coal-power plant would always be coded high-carbon). It remains outside the scope of this analysis to further differentiate ‘supporting the status quo’ category according to country-specific baselines as some other studies in existing literature have done (Vivid Economics, 2021).

An important limitation of our proposed categorisation remains that country-specific contexts of specific rescue and recovery measures can only marginally be considered given the use of general measure archetypes coding all fiscal spending measures uniformly across countries. For example, we code fiscal spending on electric vehicles as low-carbon given the relevance of electrified individual transport for a low-carbon transition. Given the need for parallel decarbonization of energy and transport, we expected that these investments will have an emission-reducing effect in the medium to long run. However, we do not further differentiate other country-specific factors such as the current and projected emission intensity of electricity supply under current policies.

#### Emissions impact type categorisation

In their analysis of stimulus spending after the financial crisis of 2009/2010, Strand and Toman (2010) argue that the categorisation of spending measures into high-carbon and low-carbon measures lacks precision to identify differences in the way that each type of spending measure affects GHG emissions over time. A more nuanced framework to assess the expected emissions impact of stimulus spending thus can enhance the understanding of how rescue and recovery ultimately affect countries’ emissions trajectories.

**Fig. 1:**
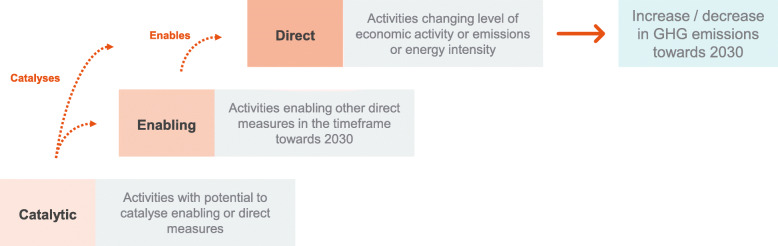
Conceptual differentiation between direct, enabling, and catalytic measures. Source: authors

For this purpose, we introduce an emissions impact type categorisation to assess the expected impact of fiscal rescue and recovery measures on GHG emissions in the period towards 2030 (Fig. 1). The proposed impact type categorisation differentiates between ‘direct’ measures, ‘enabling’ measures, and ‘catalytic’ measures. This differentiation is inspired by other existing assessment frameworks. For example, the EU Taxonomy Regulation classifies economic activities with substantial contributions to climate change mitigation targets either based on their own performance or by enabling other activities to provide substantial contributions (TEG, 2020).^2^

##### Direct emissions impact measures

We define measures as ‘direct’ if their implementation results in emissions impact due to the changes in activity levels and/or emission intensity in the timeframe towards 2030. A ‘direct’ measure’s own performance impacts GHG emissions levels. Measures that support transitional activities (for example measures that support ‘champion’ sectors or aim to boost export industries) will only be considered as ‘low-carbon’ if they unambiguously support energy or emissions efficiency improvements (TEG, 2020). Our assessment of ‘direct’ emissions impact measures also incorporates those that support ‘high-carbon’ activities, understood as those that undermine long-term environmental goals by contributing to higher GHG emissions and lead to lock-in of high-carbon infrastructure.

##### Enabling measures with no (or limited) own direct impact

We define measures as ‘enabling’ if their realisation enables the implementation of other activities with direct emissions impact in the timeframe towards 2030. An enabling measure’s own performance has no or only limited direct emission impact itself. ‘Enabling’ measures would further need to meet Do No Significant Harm (DNSH) conditions to qualify as low-carbon measures. This follows the EU Taxonomy Regulation (TEG, 2020) that stipulates that measures should (1) not lead to lock-in of assets that undermine environmental goals and (2) have a substantial positive environmental impact based on life-cycle considerations. ‘Enabling’ measures that qualify as high-carbon measures violate (or do not meet) DNSH conditions per definition.

##### Catalytic measures with no (or limited) own direct impact

We define measures as ‘catalytic’ if their implementation in the timeframe towards 2030 holds the potential to allow new direct or enabling measures to be implemented after 2030. Such ‘catalytic’ measures are characterised by uncertainty to which extent direct or enabling measures will be realised in the longer term. The role of innovation to achieve global environmental targets and provide significant economic benefits in terms of economic growth and employment is widely acknowledged (Aghion et al., 2014; O’Callaghan & Murdock, 2021). ‘Catalytic’ measures such as spending in research and development (R&D), demonstration projects, and other measures that support innovation in uncertain, yet potentially promising low-carbon technologies are characterised by their (high) uncertainty to lead to successful outcomes and their (high) potential impact to further develop emerging and immature technologies. Catalytic measures can support activities with direct or enabling impact while showing substantial time lags between investment and output (O’Callaghan & Murdock, 2021).

#### Framework combining emissions impact type and level of greenness categorisations

**Fig. 2:**
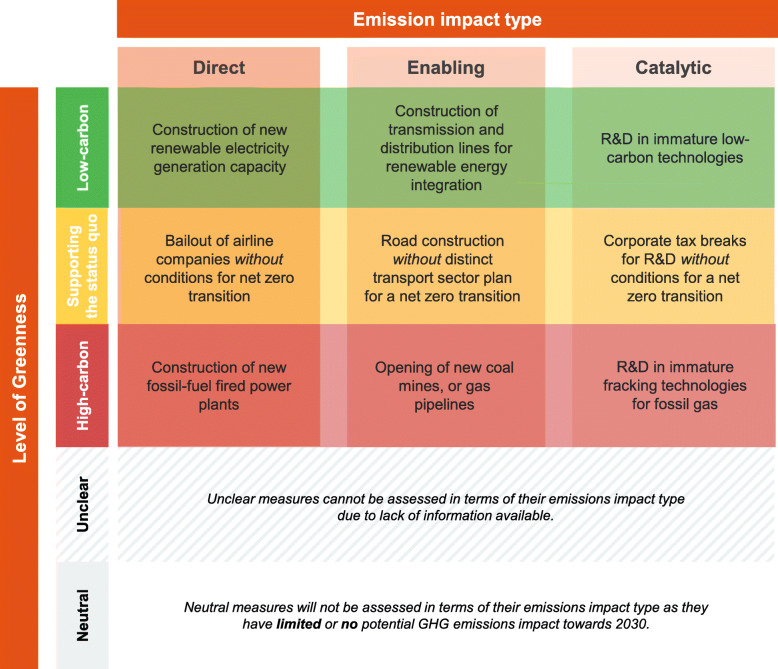
Framework to assess fiscal rescue and recovery measures according to their emissions impact type greenhouse gas emissions (GHG) towards 2030 (direct, enabling, catalytic) and their level of greenness (low-carbon, supporting status quo, high-carbon, unclear, neutral). Hypothetical examples added for illustration

The combination of both categorisations creates a typology of nine distinct measure types (see Fig. 2, complemented with hypothetical examples for illustration). Table S1 in the Supplementary Online Material (SOM) provides a detailed overview of all measure archetypes used for the subsequent analysis according to the respective assignment (see both columns on the right side in Table S1).

### Data collection and harmonisation

Our analysis primarily relies on data from the Global Recovery Observatory database as the most comprehensive fiscal rescue and recovery spending database for all key emitters except the 16 EU Member States (O’Callaghan et al., 2021b). Data from the Energy Policy Tracker fills identified data gaps for India, Japan, Indonesia, Saudi Arabia, South Africa, and Brazil (Energy Policy Tracker, 2021). Table 2 provides a complete overview of fiscal rescue and recovery databases used for this study.

**Table 2:** Overview of fiscal rescue and recovery spending databases used for this study’s data collection

Tracker (Last update)	Country coverage	Coverage of measures			Evaluation framework	
		Fiscal measures		Regulatory measures	Level of greenness	Emissions impact type
		Rescue	Recovery			
Global Recovery Observatory (O’Callaghan et al., 2021a) [Cut-off date: 28.05.2021]	50 largest economies	Yes	Yes	No	5-point Likert scale per policy (sub-)archetype used to categorise measures across countries: − 2 to + 2	5-point Likert scale per policy archetype used to categorise measures across countries on short-term GHG impact, long-term GHG impact, and net GHG impact
Energy Policy Tracker (Energy Policy Tracker, 2021) [Cut-off date: 01.06.2021]	G20 plus 11 additional countries	Yes	Yes	Yes	Coding of single measures as *conditional* or *unconditional* support for energy types: (1) fossil, (2) clean, (3) other	*Not available*
Green Recovery Tracker (E3G & Wuppertal Institut, 2021) [Cut-off date: 01.06.2021]	16 EU Member States	No	Yes	No	Coding of single measures on a 6-point Likert scale to assess the level of contribution to the green transition: (1) neutral, (2) unclear, (3) very negative, (4) negative, (5) positive, and (6) very positive	*Not available*
This study	10 key emitters plus 16 EU Member States	Yes	Yes	Partially	Coding per policy (sub-) archetype used to categorise measures across countries: (1) high-carbon, (2) supporting status quo, (3) low-carbon, (4) unclear, and (5) neutral	Coding per policy (sub-) archetype used to categorise measures across countries: (1) direct, (2) enabling, and (3) catalytic

Data for the 16 EU Member States comes from the Green Recovery Tracker database (E3G & Wuppertal Institut, 2021), including domestically and EU-funded fiscal rescue and recovery measures.^3^ This dataset, however, excludes tracking of any liquidity support provided to corporations and other rescue measures, focusing entirely on recovery spending. For this reason, a direct comparison of 16 EU Member States assessed in this analysis against other key emitters faces certain limitations.

We coded all rescue and recovery measures from the three datasets using the Global Recovery Observatory’s archetype list (O’Callaghan, 2021) and systematically checked all data entries to match the assigned archetype (a total of 2472 measures). The list has been extended to include the level of greenness and emissions impact type of our framework introduced in Section 0 (see full archetype list in Table S1 of the SOM).

The first step in the coding process was to take the mentioned archetype list of possible fiscal stimulus spending measures and assign a level of greenness and emissions impact type code to each archetype. In a second step, we applied this code to the Global Recovery Observatory’s dataset which already included a categorisation of measures according to their own typology. We then applied this typology to measures from the Green Recovery Tracker and the Energy Policy Tracker and cross-checked the data for each country to avoid double counting between different trackers (where applicable). Measures for which we identified likely double counting but could not be confirmed due to lack of precise information were excluded from the count. In the last step, we performed a systematic review of individual measures to check for coding misfits, i.e. measures for which our initial coding per policy archetype does not properly fit the individual measure. Table S2 of the SOM provides further information on the data collection, data harmonisation, and data coding.

## Results and discussion

**Fig. 3:**
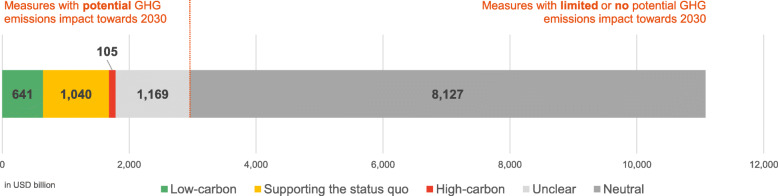
Overview of aggregated fiscal rescue and recovery spending for 10 key emitting countries and 16 Member States of the European Union assessed in the present analysis as of May 2021. Source: Data from Global Recovery Observatory (2021) and Energy Policy Tracker (2021) for ten key emitters and Green Recovery Tracker (2021) for 16 EU Member States with authors’ own coding of all individual measures inspired by measure archetype of the Global Recovery Observatory (O’Callaghan, 2021)

The ten selected key emitters and 16 Member States of the European Union (16 EU MS thereafter) jointly commit to a total of around USD 11.1 trillion in fiscal rescue and recovery spending as of May 2021 (Fig. 3). Of this, we identify around USD 3 trillion as fiscal spending with potential GHG emissions impacts for further analysis on its likely emissions impact. The other USD 8.1 trillion represent ‘neutral’ fiscal spending that no potential GHG emissions impact in line with our framework introduced in the ‘Categorisation of fiscal rescue and recovery measures by their level of greenness and their emission impact type’ section.

### Level of greenness across key emitters’ fiscal spending

**Fig. 4:**
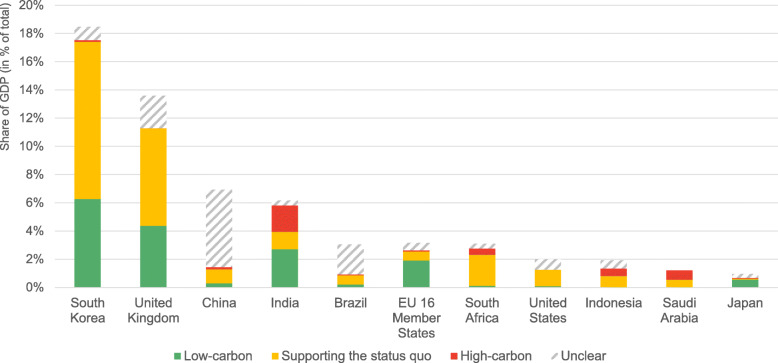
Fiscal rescue and recovery spending as of May 2021 with potential greenhouse gas (GHG) emissions impact per key emitter as a share of GDP

Most countries dedicate fiscal rescue and recovery spending to measures considered ‘supporting the status quo’ to further accelerate climate action (Fig. 4), representing an average of 35% across all countries and up to 73% of fiscal spending for single countries. This spending includes a wide range of measures such as liquidity support for large corporations or general value added tax (VAT) reductions without any conditions for a net zero transition.

Large shares of fiscal rescue and recovery spending further remain ‘unclear’ given lack of detailed information, totalling to almost USD 1.2 trillion across all countries (40% of all fiscal spending with potential impact). This is especially relevant for large fiscal spenders such as the 16 EU MS, the USA, China, the UK, and India. In the case of China, this is for example driven by lack of granularity in China’s 2021 Government Work Report announced in March 2021 available to the authors (O’Callaghan et al., 2021b).

The share of low- and high-carbon fiscal spending differs among the key emitters analysed (Fig. 4), both in absolute terms and relative to GDP. Low-carbon measures represent around USD 641 billion (22% of all spending with potential GHG emissions impact). This finding is in line with the existing literature; O’Callaghan and Murdock estimated that 18.0% of all recovery spending of the 50 largest economies could be considered low-carbon (O’Callaghan & Murdock, 2021); the Green Recovery Tracker estimated that 30% of spending assessed positive (16%) or very positive (14%) in Green Recovery Tracker’s briefing for 16 EU Member States (Green Recovery Tracker, 2021); only 17% to 19% of a total USD 2.25 trillion in announced COVID-19 ‘recovery’ spending as of May 2021 has gone towards green spending, and only 2.5% to 12.1% of total COVID-19 spending has been green or with green co-benefits (O’Callaghan, 2021; OECD, 2021; Vivid Economics, 2021).

The UK, South Korea, Japan, India, and the 16 EU Member States show higher shares of low-carbon spending (30% or more of all rescue and recovery spending with potential GHG emissions impact). Except for Japan, these countries have also committed the highest total amounts of fiscal rescue and recovery spending. Measures explicitly considered high-carbon amount to USD 105 billion (~ 4% of all spending) across all countries. India (30% of its total spending) together with Saudi Arabia (56%) and Indonesia (27%) also spent the highest shares of their domestic rescue and recovery spending on high carbon measures.

The summary results across key emitters show that around 35% of fiscal spending reinforces a current status quo and have not met the pledges to effectively focus economic rescue and recovery measures on low-carbon activities. South Korea and the UK dedicate large shares of their rescue and recovery spending to measures supporting the status quo in their economies, representing 11% and 8% of their GDP, respectively. Across all countries, these measures comprise corporate liquidity support for large corporates (total of USD 212 billion) and airline companies (USD 138 billion), reduction in interest rates (USD 183 billion), road construction (USD 90 billion), or VAT reductions (USD 53 billion)—all without specific conditions for a low-carbon transition or a specific focus on low-carbon products. This suggests that governments might have pursued other socio-economic considerations, especially during the initial rescue phase, and showed limited capabilities or willingness to align all emission-relevant fiscal spending with the Paris Agreement’s objectives.

On a positive note, explicitly low-carbon spending (22%) outweighs high-carbon spending roughly five to one. High uncertainty and a lack of available information remains on many rescue and recovery measures given that unclear spending represents around 40% of all relevant spending with potential GHG emission impact.

### Emissions impact type across key emitters’ low-carbon and high-carbon fiscal spending

**Fig. 5:**
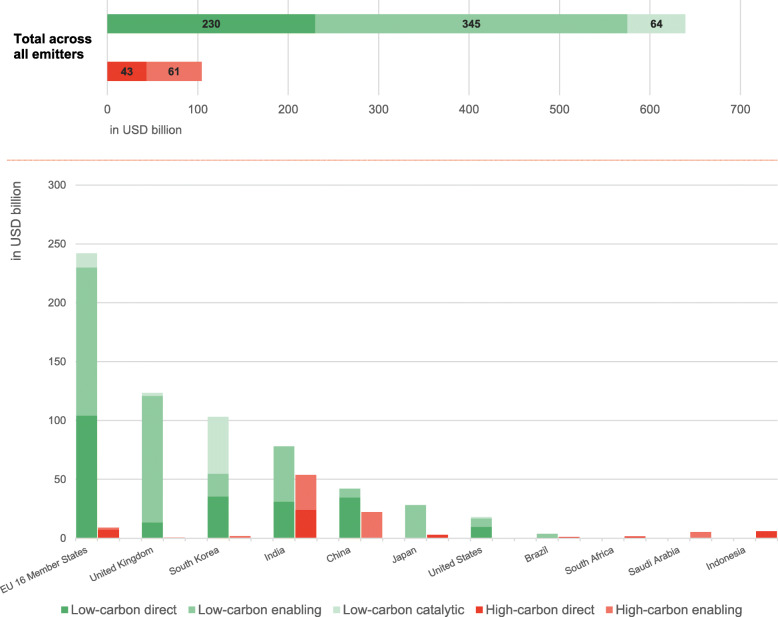
Low-carbon and high-carbon fiscal rescue and recovery spending per key emitter as of May 2021 differentiated by emissions impact type in USD billion

The emission impact type categorises fiscal rescue and recovery measures according to their expected likely impact on GHG emissions in the period towards 2030. Table 3 in the Appendix introduces several examples for rescue and recovery measures considered direct, enabling and catalytic in this study based on the harmonised dataset of almost 2500 measures. Our analysis across key emitters suggests that most of the low-carbon fiscal spending identified will likely not lead to direct emissions reductions in the short-term as almost two-thirds of the low-carbon spending of a total USD 641 billion can be considered enabling and catalytic low-carbon measures (Fig. 5). The other one-third of all low-carbon spending goes to direct low-carbon measures. Our findings suggest low-carbon fiscal rescue and recovery spending to date will likely unfold its emission reduction impact only over a longer time horizon towards 2030 and beyond. The detailed assessment of the emission impact type of low-carbon and high-carbon measures reveals heterogeneity in spending patterns among key emitters.

In total, all key emitters have spent or announced around USD 230 billion on direct low-carbon measures, representing 36% of total low-carbon spending (USD 641 billion). Except for China (82%), USA (53%), and South Africa (100%), all countries spend less than 50% of their total low-carbon spending on direct measures.

Across all key emitters analysed, USD 345 billion have been spent or announced on low-carbon enabling measures, representing around 54% of total low-carbon spending. Some countries like the UK, Japan, or Brazil dedicate more than 80% of their low-carbon spending to enabling measures. Other countries such as India, the EU16 and the USA dedicate at least 40% of their low-carbon spending to enabling measures.

Low-carbon catalytic measures represent 10% of all low-carbon spending (USD 64 billion). South Korea (47%) represents the only country spending more than 10% of their total low-carbon spending.

Both enabling and catalytic low-carbon measures will play an important role to support the implementation of direct low-carbon measures on a longer time horizon, for example through catalytic R&D and enabling infrastructure investments but might not immediately lead to the implementation of direct measures itself in the short run. Substantial further action will be required to effectively reduce emissions globally in the short to medium run towards 2030.

As for high-carbon spending of USD 104 billion across key emitters, around 42% of all high-carbon spending can be considered direct in nature, with 58% representing high-carbon enabling spending.

A total of USD 43 billion has so far been spent or announced in all key emitters analysed for high-carbon direct measures, representing 42% of all high-carbon spending. Five countries have dedicated all of their high-carbon spending to direct measures, including the UK, Japan, and Brazil. Other countries such as 16 EUMS (79%) and India (45%) have also partially spent on high-carbon direct measures.

High-carbon enabling measures constitute the remaining 58% (USD 61 billion) of high-carbon spending. The USA (100%), South Korea (100%), China (100%), and Saudi Arabia (96%) have dedicated all or almost all their high-carbon spending to high-carbon enabling measures. India (55%), and 16 EU MS (19%) have also spent partially on high carbon enabling measures, while other countries register no spending on this category.

### Policy implications of the findings

Our analysis based on the publicly available data as of May 2021 suggests that key emitters collectively may have missed the opportunity to use their fiscal rescue and recovery spending to build back their economy while fully making good on their responsibility to implement a ‘green’ recovery aligned with the Paris Agreement goals. The share of low-carbon spending across all key emitters assessed accounts for only 22% of all spending with potential GHG emissions impact. Governments might still have some leeway to adjust some parts of the fiscal spending not considered as low-carbon (high-carbon, unclear, supporting the status quo), for example by repurposing committed funding to low-carbon activities. Some countries assessed in this study show that such realigned spending can be done. However, they would need to do so in a timely manner.

Explicitly low-carbon spending outweighs high-carbon spending roughly five to one. This generally supports the need to increase the share of low-carbon spending in direct comparison to high-carbon spending to align investments with the Paris Agreement, for example overtaking high-carbon investments in the energy sector globally by around 2025 or before and growing thereafter (McCollum et al., 2018). This, however, has to put into context of around 75% of all rescue and recovery spending either remains unclear (40%) or substantiates current business-as-usual practice in the respective country context (35%).

Another important implication of this study’s findings is that two-thirds of the total low-carbon spending was identified to be of enabling and catalytic nature. This implies that the emission impact of these expenditures would only unfold over a longer time horizon. While global CO_2_ emissions already seem to have almost fully rebounded to pre-crisis levels of 2019 after experiencing the largest annual percentage decline since World War II (IEA, 2021a, 2021b), it remains to be seen whether and to what extent these expenditures would have an impact on emissions toward 2030 and beyond. Beyond the scope of this analysis, policy makers and researchers ought to enhance the empirical knowledge base on how the fiscal response to the COVID-19 pandemic can be understood in the context of transformational change imperative towards a low-carbon economy, and how national circumstances and barriers influence the process from implemented measures to actual impact on the ground.

While governments’ spending decisions in the context of the COVID-19 economic recovery consider many important national and socio-economic circumstances facing a global health and economic crisis, their collective actions remain inadequate considering both the scientific evidence on the urgency to fight climate change and governments’ own long-term climate commitments.

### Limitations and avenues for further research

The analysis for key emitters faces several methodological limitations. First, the refined framework and its application to a large dataset of fiscal rescue and recovery measures across several emitters neither measures the anticipated impact on emissions nor considers the process and timing of transformation in each country context. It allows to classify fiscal measures in terms of their causal effect on emissions but does not provide an underlying theory of transformational change for different types of fiscal rescue and recovery spending. Country-specific contexts crucially matter in several dimensions to assess the latter, for example, specific measure designs, existing barriers, or enabling factors to determine the likely GHG impact on the ground. Such analysis remains outside the scope of this analysis.

The second relates to the data cut-off date; the analysis considered all fiscal rescue and recovery announcements for the key emitters as of May 2021. For this reason, the present analysis only represents a snapshot in time for all key emitters assessed. Moreover, some of the reported response measures included in our analysis are still pending approval: the tracking of fiscal spending of EU Member States by May 2021 built on draft versions of their Recovery & Resilience Plans pending further adjustments final approval by national governments and the Council of the European Union as of May 2021. At the same time, national governments’ fiscal measures may become less driven by the responses to the pandemic as the world gradually shifts toward a post-COVID ‘new normal’. In this regard, we argue that the findings of this study are representative of countries’ fiscal measures, both implemented and planned or under discussion, primarily driven by the pandemic. Data for the EU Member States comes exclusively from the Green Recovery Tracker (E3G & Wuppertal Institut, 2021). This tracker is limited to recovery measures only and exclusively covers EU member states. This difference in scope limits the comparability of results between the 16 EU Member States and the other key emitters covered in this study. As of May 2021, the Green Recovery Tracker only provided data for 16 EU Member States (out of 27 EU Member States in total), not all 27 EU Member States, which determined the selection of the 16 EU Member States. This selection, however, covers all G20 members within the EU (Germany, France, Italy) and other larger economies (Spain, Poland), representing 85% of the EU’s GHG emissions excluding land use in 2019 (Gütschow et al., 2021). Member States left out and with slightly different spending patterns (for example Denmark, The Netherlands, or Sweden with potentially higher shares of low-carbon spending than the average EU Member State) might bias the results for EU Member States to some degree, but not change the overall findings across all emitters analysed.

Third, there was limited or no information on the timeframe of announced investments and the total committed amount for several rescue and recovery measures. For example, only a unitary subsidy value is provided for some measures providing subsidies for activities such as the purchase of electric vehicles, but no estimate of the programme’s total budgetary scope. Furthermore, many measures were announced as part of larger rescue and recovery packages, where a disaggregation of the announced expenditures committed per individual measure was not available. Following the approach by O’Callaghan (2021), we have assumed an even split between measures in some of these cases. Still, we do not have information on the amount committed for 518 measures (18% of measures collected in the database for key emitters analysed). While this uncertainty would not affect the overall findings of our analysis due to a relatively small share of total fiscal spending, any interpretation of our country-level results should consider this limitation.

Fourth, we experience a lack of granularity in applying measure archetypes to a diverse range of rescue and recovery measures with country-specific contexts. Policy options available to governments across the world are similar enough that the application of standardised policy archetypes to categorise public spending allows for a meaningful comparison across countries. However, using such an archetype to code the level of greenness and the emissions impact type of policies across countries may potentially ignore country-specific contexts. For example, the GHG emission impact of electric vehicle investments can be substantially affected by the electricity mix of the country where these investments are rolled out. In other cases, case-by-case judgements had to be made to assign a level of greenness to a rescue and recovery measure archetype: For example, measures catalogued as “other building upgrade support” included green components such as support for “eco-friendly facilities and schools”, while in other cases measures under the same archetype included traditional building upgrades or maintenance investments. In total, we have manually recoded around 100 measures to account for measure-specific information.

Fifth, we encounter limits to the extent we can harmonise the three different datasets with substantial differences in scopes. The basis for our analysis is the Global Recovery Observatory database (O’Callaghan et al., 2021a), which includes both rescue and recovery measures focusing on fiscal spending. This data is used for every country covered except the 16 EU Member States. For six key emitters analysed, we fill identified data gaps in the Global Recovery Observatory database using data from the Energy Policy Tracker (2021). This database exclusively tracks measures that support the energy sector, both fossil and low-carbon energy, and it covers both rescue and recovery measures. While harmonising data from the Energy Policy Tracker with the Global Recovery Observatory database helps to provide a more complete picture, this process required systematic cross-checking to avoid double-counting.

Considering these limitations, we identify two key avenues for future research. First, further research can conceptually embed the fiscal spending—both for regular fiscal budget cycles and fiscal rescue and recovery spending—into the literature on theories of transformational change. Such research substantiates the conceptual understanding of the process of transformation that fiscal spending can contribute to and identify the relevant conditions and barriers determining impact. Second, in-depth country assessments can complement the present cross-country analysis in several meaningful ways empirically assessing the likely or actual impact on transformation processes and emissions over time. A deeper understanding of the specific country contexts allows to better circumvent challenges in data collection and harmonisation outlined above.

## Conclusions and recommendations

More than 18 months into the COVID-19 pandemic, global CO_2_ emissions seem to have almost fully rebounded to pre-crisis levels of 2019 after experiencing the largest annual percentage decline since World War II (IEA, 2021a, 2021b). Despite early announcements by governments to prioritise an inclusive and low-carbon recovery, our analysis of 16 EU Member States and 10 selected key emitters suggests that only parts of governments’ fiscal stimulus spending indeed has been allocated to low-carbon measures (22% across all countries assessed). Instead, they dedicate large shares of their rescue and recovery spending to measures supporting the status quo in their economies (35%) and even provide some fiscal resources to explicitly high-carbon measures (4%). Our findings also suggest that countries differ substantially in terms of measures considered ‘supporting the status quo’, going up to 11% of GDP in the case of South Korea. Governments would have had the chance to implement robust conditions or incentives for a low-carbon transition as part of these measures to further support green recovery.

While low-carbon spending is significant in size across countries (USD 641 billion), almost two-thirds of it is not expected to lead to direct emissions reductions towards 2030. This spending rather supports measures considered enabling (54% of low-carbon spending) or catalytic (10%) in nature and might rather unfold its impact over time. These findings suggest that the low-carbon spending’s causal effect on GHG emissions will rather unfold over time, given that low-carbon measures categorised to have a direct emission reduction impact only represented around one-third of total low-carbon spending across countries.

We draw three recommendations for policy makers from our analysis. First, governments worldwide should enhance their preparedness and capabilities to design and swiftly implement low-carbon rescue and recovery measures in a timely manner after experiences with both the Global Financial Crisis in 2009/2010 and the outbreak of the COVID-19 pandemic in 2020. Across all countries, for example, measures supporting the status quo comprise corporate liquidity support for large corporates and airline companies, road construction, or VAT reductions—all without specific conditions for a low-carbon transition or a specific focus on low-carbon products. Policy makers can put regulatory frameworks in place and enhance their capacities to define key considerations for low-carbon transition for any such measures in times of crisis. Such forward-looking capacity building, both in developed and developing countries, can provide effective short-term rescue and recovery spending while aligning fiscal flows with sectoral transitions to net zero emissions.

Second, this also points to the overall importance of well-developed project pipelines in line with both national mid- and long-term targets and the Paris Agreement objectives. This way governments enhance their flexibility to identify appropriate low-carbon measures across different sectors in the economy in a timely manner once economic and social crises arise.

Third, the identification of key lessons learnt in the policy response to the COVID-19 pandemic remains important to inform streamlining of low-carbon budgeting into upcoming annual fiscal budgeting cycles and investment projects. While fiscal spending in response to the pandemic has been unprecedented at scale, reaching around USD 11.1 trillion in fiscal rescue and recovery spending as of May 2021 for the countries assessed in this study, upcoming fiscal budgeting cycles will matter even more so given tight limited resources after months into the pandemic.

## Supplementary Information

**Additional file 1.** S1: Data collection and data coding. Table S1: Overview of datasets from Global Recovery Observatory (2021), Energy Policy Tracker (2021), and Green Recovery Tracker (2021) used for key emitters analysed. Table S2: Overview of policy archetypes used in this study to assess rescue and recovery measures in terms of level of greenness and emissions impact type, adapted from O’Callaghan and Murdock (2021, see Appendix A).

## Data Availability

All datasets used for the analysis are publicly available and are provided in the bibliography. Supplementary online material attached to the manuscript provides codebook used for the analysis and other relevant materials.

## Appendix

**Table 3:** Selected examples of rescue and recovery measures under different impact type categories (‘low-carbon direct’, ‘low-carbon enabling’, ‘low-carbon catalytic’, ‘high-carbon direct’, and ‘high-carbon enabling’)

Measure	Type (level of greenness and emissions impact type)	Explanation
GBR-OXF-016: Net Zero Innovation Portfolio	Low-carbon catalytic	• GBP 200 million to stimulate private sector investment into near-to-market low-carbon energy innovations, including Direct Air Capture and green hydrogen. • **Explanation of coding decision for this study:** Investment in relatively mature new technologies, focusing on cost-efficiency improvement for market competitivity could potentially have moderate/big payoff in mid-term.
KOR-OXF-107: Green Innovation	Low-carbon catalytic	• USD 2.35 billion for R&D in green technologies focusing on long-term support schemes for technology development. Part of a series of measures supporting green innovation in the industry sector. • **Explanation of coding decision for this study:** Long-term support for new green technologies can catalyse growth of the sector in the mid to long term.
CHN-OXF-032: Green Vehicle Investment	Low-carbon enabling	• Investments in electric vehicle battery charging/swapping facilities, and battery recycling infrastructure as part of the CNY 2.8 trillion 2021 Government Work Report. Aims to contribute to develop the necessary charging infrastructure for the expansion of low-carbon transport. • **Explanation of coding decision for this study:** Development of necessary infrastructure for competitive green technologies can boost its expansion in the mid-term.
BRA-EPT-035: BNDES financing of wind generator blades	Low-carbon enabling	• USD 10 million for national manufacturer of wind generator blades. • **Explanation of coding decision for this study:** Measure aiming at boost national wind industry and enable a quicker roll-out of wind power in the medium-term, provides necessary supporting infrastructure for already competitive technologies.
IND-OXF-054: Support to coal mining sector	High-carbon enabling	• USD 6.8 billion to develop coal transportation infrastructure as part of a plan to expand domestic production and replace imports. • **Explanation of coding decision for this study:** Measure enables higher domestic coal production and might support further lock-in of coal power infrastructure.
SAU-EPT-001: Hawiyah Unayzah underground gas storage site	High-carbon enabling	• USD 1.85 billion on underground gas storage facility. • **Explanation of coding decision for this study:** Measure supports the expansion of fossil gas production and further locks in fossil fuel power infrastructure.
GBR-OXF-101: new road projects	‘Supporting the status quo’ enabling	• USD 35 billion to be spent until 2025 on developing road infrastructure • **Explanation of coding decision for this study:** Measure supports the expansion of traditional transport infrastructure without specific consideration of a low-carbon transition and aligned plans to reduce emissions from the transport sector, enabling an increase in future transport activity.
JPN-EPT-008: project to advance RE and EVs simultaneously	Low-carbon direct	• USD 80 million for short-term and intensive support for the deployment of EVs, fuel cell vehicles along with the spread of RE, promoting a zero-carbon lifestyle. • **Explanation of coding decision for this study:** Measure supports am increase in RE power generation and switch to ULEVs which directly impact emissions from electricity and transport.
ZAF-EPT-016: procurement of new generation capacity from gas, diesel, coal and storage	High-carbon direct	• National plans for new generation capacity through 513 MW from storage (for the year 2022), 3000 MW from gas. (for the years 2024 to 2027) and 1500 MW from coal (2023 to 2027) • **Explanation of coding decision for this study:** Support for new fossil fuel power generation contributes to direct GHG emission increases.
BRA-OXF-014: Unconditional airline support	‘Supporting the status quo’ direct	• USD 650 million rescue package for major Brazilian airlines negotiated with BNDES without any green conditions • **Explanation of coding decision for this study:** Measure considered ‘supporting the status quo’ given no specific conditions for a low-carbon transition of supported airlines required, while airlines will continue their activity straight away (direct).

## Footnotes

1 EU Member States included in the analysis are Austria, Belgium, Bulgaria, the Czech Republic, Estonia, Finland, France, Germany, Italy, Latvia, Poland, Portugal, Romania, Slovenia, Spain, and Slovakia.

2 Within our definition of ‘direct’ emissions impact measures, we include measures that support activities considered to contribute based on their own performance (i.e. those associated with sequestration or very low or zero emissions) and those considered in the taxonomy as transitional activities (i.e. those activities considered necessary in a zero-carbon economy, but which are not currently close to a zero-carbon level).

3 Our analysis assesses the full scope of rescue and recovery spending in the 16 EU Member States. This should not be confused with the sub-part of their spending from the EU Recovery and Resilience Facility (RRF), which imposes a minimum of 37% to be spent on climate change and applies a ‘do-no-significant-harm’ principle to all EU RRF funding.
