# Supplementary material for: Unpacking the COVID-19 rescue and recovery spending: an assessment of implications on greenhouse gas emissions towards 2030 for key emitters
Source: Clim Action. 2022 Mar 7;1(1):3. doi: 10.1007/s44168-022-00002-9 (PMC8900090; doi:10.1007/s44168-022-00002-9)
Supplement: Supplementary file 1 — Additional file 1. S1: Data collection and data coding. Table S1: Overview of datasets from Global Recovery Observatory (2021), Energy Policy Tracker (2021), and Green Recovery Tracker (2021) used for key emitters analysed. Table S2: Overview of policy archetypes used in this study to assess rescue and recovery measures in terms of level of greenness and emissions impact type, adapted from O’Callaghan and Murdock (2021, see Appendix A). [file 44168_2022_2_MOESM1_ESM.docx]

**Supplementary Online Material to:**

**Unpacking the COVID-19 rescue and recovery spending: an assessment of implications on greenhouse gas emissions towards 2030 for key emitters**

**Table of Contents**

[S1: Data collection and data coding 2](#_Toc93937856)

[Data collection 2](#_Toc93937857)

[Data coding 3](#_Toc93937858)

[References 11](#_Toc93937859)

S1: Data collection and data coding

## Data collection

The dataset by the Global Recovery Observatory (2021) presents the main data source for all ten key emitters, except for the 16 Member States of the European Union. We complement the Global Recovery Observatory (2021) dataset with data from the Energy Policy Tracker (2021) for six countries for which there have been data gaps identified (Japan, India, Brazil, South Africa, Indonesia, Saudi Arabia).

For 16 Member States of the European Union, we use the dataset by the Green Recovery Tracker (2021) that considers national-level measures funded both from domestic budgets and EU-level budgets.^[[1]](#footnote-2)^ As of May 2021, the Green Recovery Tracker (2021) has no other data available for the remaining 11 Member States of the European Union.

Table S1 provides the data collection for all key emitters analysed for this analysis as of May 2021.

Table S1: Overview of datasets from Global Recovery Observatory (2021), Energy Policy Tracker (2021), and Green Recovery Tracker (2021) used for key emitters analysed.

|  | Global Recovery Observatory | Energy Policy Tracker | Green Recovery Tracker |
| --- | --- | --- | --- |
| Brazil | **Yes**,  as main dataset | **Yes**,  for complementing data set | [-] |
| China | **Yes**,  as main dataset | [-] | [-] |
| 16 EU Member States | **Yes**,  used for cross-checking data in *Green Recovery Tracker* | [-] | **Yes**,  as main dataset |
| India | **Yes**,  as main dataset | **Yes**,  for complementing data set | [-] |
| Indonesia | **Yes**,  as main dataset | **Yes**,  for complementing data set | [-] |
| Japan | **Yes**,  as main dataset | **Yes**,  for complementing data set | [-] |
| Saudi Arabia | **Yes**,  as main dataset | **Yes**,  for complementing data set | [-] |
| South Africa | **Yes**,  as main dataset | **Yes**,  for complementing data set | [-] |
| South Korea | **Yes**,  as main dataset | [-] | [-] |
| United Kingdom | **Yes**,  as main dataset | [-] | [-] |
| USA | **Yes**,  as main dataset | [-] | [-] |

## Data coding

We use the sub-archetypes for rescue and recovery measures introduced by the Global Recovery Observatory (O’Callaghan & Murdock, 2021, Appendix A) to assign the level of greenness and emissions impact type per sub-archetype (Table S2).

All measures collected by the Global Recovery Observatory (2021) have a sub-archetype assigned in the dataset. We have manually reviewed every single measure and re-assigned 154 measures. Measure packages where a part of the budget remains *unallocated* have been coded either as ‘*neutral*’ (in cases where the package itself had a scope that lies outside of our analysis, such as job protection schemes) or as ‘*unclear*’ (in cases where the package might include measures within the scope of the analysis, for example support for large businesses or infrastructure investment packages).

For measures reported in other data sources, we have manually coded a total of 156 measures listed in the Energy Policy Tracker (2021) for six countries of Japan, India, Brazil, South Africa, Indonesia, and Saudi Arabia. We also manually coded a total of 936 measures listed in the Green Recovery Tracker (2021) for 16 Member States of the European Union.

Table S2: Overview of policy archetypes used in this study to assess rescue and recovery measures in terms of level of greenness and emissions impact type, adapted from O’Callaghan and Murdock (2021, see Appendix A) .

| Code | Archetype | Short-term GHG emissions (SE) | Long-term GHG emissions (LE) | Type#1 | Level of greenness | Emissions impact type |
| --- | --- | --- | --- | --- | --- | --- |
| A Indiscriminate | Liquidity support for subnational public entities | **SE** | **LE** | Rescue | Unclear | Direct |
| A1 | Support for states/regions | **-1** | **0** | Rescue | Unclear | Direct |
| A2 | Support for localities | **-1** | **0** | Rescue | Unclear | Direct |
| B Indiscriminate | Liquidity support for large businesses | **SE** | **LE** | Rescue | Supporting the status quo | Direct |
| B1 | Support for agriculture, forestry, and fishing (no green conditions) | **-1** | **0** | Rescue | Supporting the status quo | Direct |
| B2 | Support for agriculture, forestry, and fishing (with green conditions) | **-1** | **1** | Rescue | Low-carbon | Direct |
| B3 | Support for airlines and other transport (no green conditions) | **-2** | **0** | Rescue | Supporting the status quo | Direct |
| B4 | Support for airlines and other transport (with green conditions) | **-2** | **1** | Rescue | Low-carbon | Direct |
| B5 | Support for energy (no green conditions) | **-2** | **-1** | Rescue | High-carbon | Direct |
| B6 | Support for energy (with green conditions) | **-2** | **1** | Rescue | Low-carbon | Direct |
| B7 | Support for holiday and leisure (no green conditions) | **-1** | **0** | Rescue | Supporting the status quo | Direct |
| B8 | Support for holiday and leisure (with green conditions) | **-1** | **1** | Rescue | Low-carbon | Direct |
| B9 | Support for retail (no green conditions) | **-1** | **0** | Rescue | Supporting the status quo | Direct |
| B10 | Support for retail (with green conditions) | **-1** | **1** | Rescue | Low-carbon | Direct |
| B11 | Support for specified other industry (no green conditions) | **-1** | **0** | Rescue | Supporting the status quo | Direct |
| B8 | Support for holiday and leisure (with green conditions) | **-1** | **1** | Rescue | Low-carbon | Direct |
| B9 | Support for retail (no green conditions) | **-1** | **0** | Rescue | Supporting the status quo | Direct |
| B10 | Support for retail (with green conditions) | **-1** | **1** | Rescue | Low-carbon | Direct |
| B11 | Support for specified other industry (no green conditions) | **-1** | **0** | Rescue | Supporting the status quo | Direct |
| B12 | Support for specified other industry (with green conditions) | **-1** | **1** | Rescue | Low-carbon | Direct |
| B13 | Support for unspecified industry | **-1** | **0** | Rescue | Neutral |  |
| C Indiscriminate | **Liquidity support for startups and SMEs** | **SE** | **LE** | Rescue | Neutral |  |
| C1 | Support for agriculture, forestry, and fishing | **-1** | **0** | Rescue | Neutral |  |
| C2 | Support for energy | **-2** | **0** | Rescue | Neutral |  |
| C3 | Support for holiday and leisure | **-1** | **0** | Rescue | Neutral |  |
| C4 | Support for retail | **-1** | **0** | Rescue | Neutral |  |
| C5 | Support for specified other industry | **-1** | **0** | Rescue | Neutral |  |
| C6 | Support for unspecified industry | **-1** | **0** | Rescue | Neutral |  |
| D Indiscriminate | **Liquidity support for not for profit organisations (NFPs)** | **SE** | **LE** | Rescue | Neutral |  |
| D1 | Support for arts and culture | **0** | **0** | Rescue | Neutral |  |
| D2 | Support for social care | **0** | **0** | Rescue | Neutral |  |
| D3 | Support for education and research institutions | **0** | **0** | Rescue | Neutral |  |
| D4 | General non-profit support | **0** | **0** | Rescue | Neutral |  |
| D5 | Support for animal services | **0** | **0** | Rescue | Neutral |  |
| E Indiscriminate | **Temporary waiver of interest payments for businesses** | **SE** | **LE** | Rescue | Neutral |  |
| E1 | Commercial rent interest relief | **-1** | **0** | Rescue | Supporting the status quo | Direct |
| E2 | Automotive interest relief | **-1** | **0** | Rescue | Supporting the status quo | Direct |
| E3 | Utility payment interest relief (i.e. electricity, gas, water) | **-1** | **0** | Rescue | Supporting the status quo | Direct |
| E4 | Microcredit interest relief | **-1** | **0** | Rescue | Supporting the status quo | Direct |
| E5 | Rural investment interest relief | **-1** | **0** | Rescue | Supporting the status quo | Direct |
| E6 | General and other | **-1** | **0** | Rescue | Supporting the status quo | Direct |
| F Indiscriminate | **Direct provision of basic needs** | **SE** | **LE** | Rescue | Neutral |  |
| F1 | Nutrition support | **0** | **0** | Rescue | Neutral |  |
| F2 | Shelter support | **0** | **0** | Rescue | Neutral |  |
| F3 | Social services support | **0** | **0** | Rescue | Neutral |  |
| F4 | Utility access support | **0** | **0** | Rescue | Neutral |  |
| F5 | General and other support | **0** | **0** | Rescue | Neutral |  |
| G Indiscriminate | **Targeted welfare cash transfers** | **SE** | **LE** | Rescue | Neutral |  |
| G1 | Payments targeted to families | **-1** | **0** | Rescue | Neutral |  |
| G2 | Payments targeted to low income individuals | **-1** | **0** | Rescue | Neutral |  |
| G3 | Payments targeted to individuals (other) | **-1** | **0** | Rescue | Neutral |  |
| G4 | Indirect payments through social programs | **-1** | **0** | Rescue | Neutral |  |
| H Indiscriminate | **Job Continuation Support** | **SE** | **LE** | Rescue | Neutral |  |
| H1 | Job continuation subsidies | **-1** | **0** | Rescue | Neutral |  |
| H2 | Job continuation incentives | **-1** | **0** | Rescue | Neutral |  |
| I Indiscriminate | **Temporary waiver of interest payments for individuals** | **SE** | **LE** | Rescue | Neutral |  |
| I1 | Mortgage interest and rental relief | **-1** | **0** | Rescue | Neutral |  |
| I2 | Student debt interest relief | **-1** | **0** | Rescue | Neutral |  |
| I3 | General and other relief | **-1** | **0** | Rescue | Supporting the status quo | Direct |
| I4 | Automotive interest relief | **-1** | **0** | Rescue | Supporting the status quo | Direct |
| J Indiscriminate | **Healthcare services support** | **SE** | **LE** | Rescue | Neutral |  |
| J1 | General medical equipment/services spending (including PPE) | **-1** | **0** | Rescue | Neutral |  |
| J2 | Mental health support | **0** | **0** | Rescue | Neutral |  |
| J3 | Aged care support | **0** | **0** | Rescue | Neutral |  |
| J4 | General medical personnel support | **0** | **0** | Rescue | Neutral |  |
| J5 | Vaccine and COVID-19 research, manufacturing, and application spending | **-1** | **0** | Rescue | Neutral |  |
| K Indiscriminate | **Emergency services (disaster management) support** | **SE** | **LE** | Rescue | Neutral |  |
| K1 | Pandemic administrative support | **0** | **0** | Rescue | Neutral |  |
| K2 | Equipment procurement | **-1** | **0** | Rescue | Neutral |  |
| K3 | Infrastructure support (short-term shelters, food-stocking, water supplies) | **-1** | **0** | Rescue | Neutral |  |
| L Indiscriminate | **Income tax cuts** | **SE** | **LE** | Rescue | Neutral |  |
| L1 | Reduction in marginal rates (including increases in tax-free thresholds) | **-1** | **0** | Rescue | Neutral |  |
| L2 | Expanded deductions | **-1** | **0** | Rescue | Neutral |  |
| L3 | New tax exemptions | **-1** | **0** | Rescue | Neutral |  |
| L4 | Permitted delays in payment | **-1** | **0** | Rescue | Neutral |  |
| M Indiscriminate | **VAT and other goods and services tax cuts** | **SE** | **LE** | Rescue | Neutral |  |
| M1 | VAT reductions | **-1** | **0** | Rescue | Supporting the status quo | Direct |
| M2 | VAT deferrals | **-1** | **0** | Rescue | Supporting the status quo | Direct |
| M3 | Non-discretionary payment relief | **-1** | **0** | Rescue | Neutral |  |
| M4 | Reduced taxes for emergency medical imports | **-1** | **0** | Rescue | Neutral |  |
| N Indiscriminate | **Business tax cuts** | **SE** | **LE** | Rescue | Unclear | Direct |
| N1 | Reduction in rates | **-1** | **0** | Rescue | Supporting the status quo | Direct |
| N2 | Expanded deductions | **-1** | **0** | Rescue | Neutral |  |
| N3 | New tax exemptions for clean investments | **0** | **1** | Rescue | Low-carbon | Direct |
| N4 | New tax exemptions for general and other investments | **-1** | **0** | Rescue | Supporting the status quo | Direct |
| O Indiscriminate | **Business tax deferrals** | **SE** | **LE** | Rescue | Supporting the status quo | Direct |
| O1 | Tax deferrals for dirty industries | **-2** | **0** | Rescue | Supporting the status quo | Direct |
| O2 | Tax deferrals for other industries | **-1** | **0** | Rescue | Supporting the status quo | Direct |
| P Indiscriminate | **Reduced prices for centrally controlled products and services** | **SE** | **LE** | Rescue | Neutral |  |
| P1 | Public service payments | **0** | **0** | Rescue | Neutral |  |
| P2 | Fuel prices (oil and gas) | **-2** | **0** | Rescue | High-carbon | Direct |
| P3 | Utility prices (electricity and water) | **0** | **0** | Rescue | Supporting the status quo | Direct |
| Q Indiscriminate | **Other tax cuts and deferrals** | **SE** | **LE** | Rescue | Unclear | Direct |
| Q1 | Other tax cuts and deferrals | **-1** | **0** | Rescue | Unclear | Direct |
| R Indiscriminate | **Targeted recovery cash transfers** | **SE** | **LE** | Recovery | Neutral |  |
| R1 | Payments targeted to families | **-1** | **0** | Recovery | Neutral |  |
| R2 | Payments targeted to low income individuals | **-1** | **0** | Recovery | Neutral |  |
| R3 | Payments targeted to individuals (other) | **-1** | **0** | Recovery | Neutral |  |
| R4 | Indirect payments through social programs | **-1** | **0** | Recovery | Neutral |  |
| S Indiscriminate | **Tourism and leisure industry incentives** | **SE** | **LE** | Recovery | Neutral |  |
| S1 | Incentives for tourism | **-1** | **0** | Recovery | Neutral |  |
| S2 | Incentives for hospitality services | **0** | **0** | Recovery | Neutral |  |
| S3 | Incentives for arts and cultural activities | **0** | **0** | Recovery | Neutral |  |
| S4 | Measures to promote leisure participation | **0** | **0** | Recovery | Neutral |  |
| T Indiscriminate | **Electric vehicle incentives** | **SE** | **LE** | Recovery | Low-carbon | Direct |
| T1 | EV transfer programs | **-1** | **2** | Recovery | Low-carbon | Direct |
| T2 | EV subsidies | **-1** | **2** | Recovery | Low-carbon | Direct |
| U Indiscriminate | **Electronic appliance and efficiency incentives** | **SE** | **LE** | Recovery | Low-carbon | Direct |
| U1 | Electronic appliance specific ‘cash for clunkers’ programs | **-1** | **1** | Recovery | Low-carbon | Direct |
| U2 | Electronic appliance subsidies | **-1** | **1** | Recovery | Low-carbon | Direct |
| V Indiscriminate | **Green market creation** | **SE** | **LE** | Recovery | Low-carbon | Enabling |
| V1 | Increased clean energy market participation | **0** | **2** | Recovery | Low-carbon | Enabling |
| V2 | Modernisation and transition investments | **0** | **2** | Recovery | Low-carbon | Enabling |
| V3 | Capacity investments | **-1** | **2** | Recovery | Low-carbon | Enabling |
| W Indiscriminate | **Other incentive measures** | **SE** | **LE** | Recovery | Unclear | Direct |
| W1 | Other incentive measures | **-1** | **0** | Recovery | Unclear | Direct |
| X Indiscriminate | **Worker retraining and job creation** | **SE** | **LE** | Recovery | Unclear | Enabling |
| X1 | Green worker retraining and job creation | **0** | **2** | Recovery | Low-carbon | Enabling |
| X2 | General and other worker retraining and job creation | **0** | **0** | Recovery | Neutral |  |
| Y Indiscriminate | **Education investment (non-infrastructure)** | **SE** | **LE** | Recovery | Neutral |  |
| Y1 | Education capital and equipment | **0** | **0** | Recovery | Neutral |  |
| Y2 | Scholarship funding | **0** | **0** | Recovery | Neutral |  |
| Y3 | Staff funding | **0** | **0** | Recovery | Neutral |  |
| Z Indiscriminate | **Healthcare investment (non-infrastructure)** | **SE** | **LE** | Recovery | Neutral |  |
| Z1 | General medical investment | **0** | **0** | Recovery | Neutral |  |
| Z2 | Mental health investment | **0** | **0** | Recovery | Neutral |  |
| Z3 | Aged care investment | **0** | **0** | Recovery | Neutral |  |
| Z4 | Healthcare capital investment | **0** | **0** | Recovery | Neutral |  |
| 𝛼 Indiscriminate | **Social and cultural investment (non-infrastructure)** | **SE** | **LE** | Recovery | Neutral |  |
| 𝛼1 | Support for arts and culture | **0** | **0** | Recovery | Neutral |  |
| 𝛼2 | Support for social care | **0** | **0** | Recovery | Neutral |  |
| 𝛼3 | General and other non-profit investment | **0** | **0** | Recovery | Neutral |  |
| 𝛽 Indiscriminate | **Communications infrastructure investment** | **SE** | **LE** | Recovery | Unclear | Enabling |
| 𝛽1 | Broadband investment | **-2** | **0** | Recovery | Low-carbon | Enabling |
| 𝛽2 | Remote working infrastructure investment | **-1** | **0** | Recovery | Low-carbon | Enabling |
| 𝛽3 | Civil cybersecurity programmes | **0** | **0** | Recovery | Neutral |  |
| 𝛽4 | Implementation of digital programmes | **0** | **0** | Recovery | Neutral |  |
| 𝛾 Indiscriminate | **Traditional transport infrastructure investment** | **SE** | **LE** | Recovery | High-carbon | Unclear |
| 𝛾1 | Road construction | **-2** | **0** | Recovery | Supporting the status quo | Enabling |
| 𝛾2 | ICE engine automobile support | **-2** | **-2** | Recovery | High-carbon | Direct |
| 𝛾3 | Aviation infrastructure | **-2** | **-2** | Recovery | High-carbon | Enabling |
| 𝛾4 | Port and ship construction | **-2** | **-1** | Recovery | High-carbon | Enabling |
| 𝛾5 | Rail construction and capacity | **-2** | **0** | Recovery | Low-carbon | Enabling |
| 𝛿 Indiscriminate | **Clean transport infrastructure investment** | **SE** | **LE** | Recovery | Low-carbon | Enabling |
| 𝛿1 | New public transport systems or line expansions | **-2** | **2** | Recovery | Low-carbon | Enabling |
| 𝛿2 | Existing public transport capacity expansions | **-2** | **2** | Recovery | Low-carbon | Enabling |
| 𝛿3 | EV charging infrastructure | **-2** | **2** | Recovery | Low-carbon | Enabling |
| 𝛿4 | Public transport digitalisation efforts | **-2** | **2** | Recovery | Low-carbon | Enabling |
| 𝛿5 | Cycling and walking infrastructure | **-2** | **2** | Recovery | Low-carbon | Enabling |
| 𝛿6 | Efficiency initiatives to improve dirty transport | **-2** | **2** | Recovery | Low-carbon | Enabling |
| 𝜀 Indiscriminate | **Traditional energy infrastructure investment** | **SE** | **LE** | Recovery | High-carbon | Unclear |
| 𝜀1 | New or refurbished power plants | **-2** | **-2** | Recovery | High-carbon | Direct |
| 𝜀2 | New or refurbished refineries | **-2** | **-2** | Recovery | High-carbon | Enabling |
| 𝜀3 | New or refurbished coal mines and oil/gas fields | **-2** | **-2** | Recovery | High-carbon | Enabling |
| 𝜀4 | New or refurbished infrastructure for transport and transmission of fossil energy inputs/outputs | **-2** | **-2** | Recovery | High-carbon | Enabling |
| 𝜂 Indiscriminate | **Clean energy infrastructure investment** | **SE** | **LE** | Recovery | Low-carbon | Unclear |
| 𝜂1 | New or refurbished renewable energy generation facilities | **-2** | **2** | Recovery | Low-carbon | Direct |
| 𝜂2 | New or refurbished nuclear energy generation facilities | **-2** | **2** | Recovery | Low-carbon | Direct |
| 𝜂3 | New biofuel and other renewable fuel infrastructure | **-2** | **2** | Recovery | Low-carbon | Direct |
| 𝜂4 | Upgraded (or new) transmission infrastructure | **-2** | **2** | Recovery | Low-carbon | Enabling |
| 𝜂5 | Upgraded (or new) distribution infrastructure including smart grids | **-2** | **2** | Recovery | Low-carbon | Enabling |
| 𝜂6 | Hydrogen infrastructure | **-2** | **2** | Recovery | Low-carbon | Direct |
| 𝜂7 | Battery and storage infrastructure | **-2** | **2** | Recovery | Low-carbon | Enabling |
| 𝜂8 | Carbon capture and storage/utilisation | **-2** | **2** | Recovery | Low-carbon | Catalytic |
| 𝜂9 | Other initiatives to clean dirty energy assets | **-2** | **2** | Recovery | Low-carbon | Direct |
| 𝜃 Indiscriminate | **Local (project-based) infrastructure investment** | **SE** | **LE** | Recovery | Unclear | Enabling |
| 𝜃1 | Urban development programs | **-2** | **0** | Recovery | Unclear | Enabling |
| 𝜃2 | General new housing investment | **-2** | **0** | Recovery | Supporting the status quo | Enabling |
| 𝜃3 | Clean new housing investment | **-2** | **1** | Recovery | Low-carbon | Enabling |
| 𝜃4 | Public building investment | **-2** | **0** | Recovery | Supporting the status quo | Enabling |
| 𝜃5 | Local utility investment | **-2** | **0** | Recovery | Supporting the status quo | Enabling |
| 𝜆 Indiscriminate | **Building upgrades and energy efficiency infrastructure investment** | **SE** | **LE** | Recovery | Low-carbon | Direct |
| 𝜆1 | Green retrofitting programs (including daylighting, electricity and electrification, insultation) | **-2** | **2** | Recovery | Low-carbon | Direct |
| 𝜆2 | Rooftop solar support | **-2** | **2** | Recovery | Low-carbon | Direct |
| 𝜆3 | Other building upgrade support | **-2** | **0** | Recovery | Supporting the status quo | Direct |
| 𝜇 Indiscriminate | **Natural infrastructure and green spaces investment** | **SE** | **LE** | Recovery | Low-carbon | Direct |
| 𝜇1 | Public parks and green spaces investment | **1** | **1** | Recovery | Low-carbon | Direct |
| 𝜇2 | Tree planting and biodiversity protection | **1** | **2** | Recovery | Low-carbon | Direct |
| 𝜇3 | Ecological conservation initiatives | **1** | **2** | Recovery | Low-carbon | Direct |
| 𝜇4 | Waterway protection and enhancement | **1** | **1** | Recovery | Neutral |  |
| 𝜇5 | Agricultural Uplift | **0** | **0** | Recovery | Neutral |  |
| 𝜋 Indiscriminate | **Other large-scale infrastructure investments** | **SE** | **LE** | Recovery | Supporting the status quo | Direct |
| 𝜋1 | Large-scale urban projects | **-2** | **0** | Recovery | Supporting the status quo | Direct |
| 𝜋2 | Large-scale regional infrastructure (dams, non-coal mines, etc) | **-2** | **0** | Recovery | Supporting the status quo | Direct |
| 𝜋3 | Large-scale space infrastructure | **-2** | **-1** | Recovery | Supporting the status quo | Direct |
| 𝜎 Indiscriminate | **Armed forces investment** | **SE** | **LE** | Recovery | High-carbon | Direct |
| 𝜎1 | Arsenal funding | **-2** | **-2** | Recovery | High-carbon | Direct |
| 𝜎2 | Administration funding | **-1** | **-2** | Recovery | High-carbon | Direct |
| 𝜏 Indiscriminate | **Disaster preparedness and capacity building investment** | **SE** | **LE** | Recovery | Neutral |  |
| 𝜏1 | Future epidemic reaction capabilities | **-1** | **0** | Recovery | Neutral |  |
| 𝜏2 | Disaster-response infrastructure (shelters, food-stocking, water supplies) | **-1** | **0** | Recovery | Neutral |  |
| 𝜏3 | Anti-flood, fires, and other climate adaptation measures | **-1** | **0** | Recovery | Neutral |  |
| 𝜑 Indiscriminate | **General research and development investment** | **SE** | **LE** | Recovery | Neutral |  |
| 𝜑1 | Health and science programmes | **0** | **1** | Recovery | Neutral |  |
| 𝜑2 | Digitisation and AI programmes | **0** | **1** | Recovery | Low-carbon | Catalytic |
| 𝜑3 | Space programmes | **0** | **1** | Recovery | Neutral |  |
| 𝜑4 | General and other programmes | **0** | **1** | Recovery | Neutral |  |
| 𝜓 Indiscriminate | **Clean research and development investment** | **SE** | **LE** | Recovery | Low-carbon | Catalytic |
| 𝜓1 | Energy sector R&D programmes | **0** | **2** | Recovery | Low-carbon | Catalytic |
| 𝜓2 | Agriculture R&D programmes | **0** | **2** | Recovery | Low-carbon | Catalytic |
| 𝜓3 | Industrial R&D programmes | **0** | **2** | Recovery | Low-carbon | Catalytic |
| 𝜓4 | Other sectoral R&D programmes | **0** | **2** | Recovery | Low-carbon | Catalytic |
| B12 | Support for specified other industry (with green conditions) | **-1** | **1** | Rescue | Low-carbon | Direct |

References

Climate Action Tracker. (2020). *Pandemic recovery: Positive intentions vs policy rollbacks, with just a hint of green. Warming Projections Global Update. September 2020*. Climate Action Tracker (Climate Analytics, NewClimate Institute). https://climateactiontracker.org/documents/790/CAT_2020-09-23_Briefing_GlobalUpdate_Sept2020.pdf

E3G, & Wuppertal Institut. (2021). *Green Recovery Tracker*. https://www.greenrecoverytracker.org/

Energy Policy Tracker. (2021). *Track funds for energy in recovery packages*. https://www.energypolicytracker.org/

O’Callaghan, B., & Murdock, E. (2021). *Are We Building Back Better? Evidence from 2020 and Pathways for Inclusive Green Recovery Spending*. https://wedocs.unep.org/bitstream/handle/20.500.11822/35281/AWBBB.pdf

UNEP. (2020). *Emissions Gap Report 2020*. United Nations Environment Programme. https://www.unep.org/resources/emissions-gap-report-2019

1. France, Germany, Spain, Portugal, Bulgaria, Austria, Czech Republic, Estonia, Finland, Italy, Latvia, Poland, Romania, Slovakia, Slovenia, Belgium [↑](#footnote-ref-2)
